# Forecasting influenza in Hong Kong with Google search queries and statistical model fusion

**DOI:** 10.1371/journal.pone.0176690

**Published:** 2017-05-02

**Authors:** Qinneng Xu, Yulia R. Gel, L. Leticia Ramirez Ramirez, Kusha Nezafati, Qingpeng Zhang, Kwok-Leung Tsui

**Affiliations:** 1 City University of Hong Kong, Hong Kong SAR, China; 2 University of Texas at Dallas, Dallas, United States of America; 3 Centro de Investigación en Matemáticas, Mexico City, Mexico; University of Hong Kong, HONG KONG

## Abstract

**Background:**

The objective of this study is to investigate predictive utility of online social media and web search queries, particularly, Google search data, to forecast new cases of influenza-like-illness (ILI) in general outpatient clinics (GOPC) in Hong Kong. To mitigate the impact of sensitivity to self-excitement (i.e., fickle media interest) and other artifacts of online social media data, in our approach we fuse multiple offline and online data sources.

**Methods:**

Four individual models: generalized linear model (GLM), least absolute shrinkage and selection operator (LASSO), autoregressive integrated moving average (ARIMA), and deep learning (DL) with Feedforward Neural Networks (FNN) are employed to forecast ILI-GOPC both one week and two weeks in advance. The covariates include Google search queries, meteorological data, and previously recorded offline ILI. To our knowledge, this is the first study that introduces deep learning methodology into surveillance of infectious diseases and investigates its predictive utility. Furthermore, to exploit the strength from each individual forecasting models, we use statistical model fusion, using Bayesian model averaging (BMA), which allows a systematic integration of multiple forecast scenarios. For each model, an adaptive approach is used to capture the recent relationship between ILI and covariates.

**Results:**

DL with FNN appears to deliver the most competitive predictive performance among the four considered individual models. Combing all four models in a comprehensive BMA framework allows to further improve such predictive evaluation metrics as root mean squared error (RMSE) and mean absolute predictive error (MAPE). Nevertheless, DL with FNN remains the preferred method for predicting locations of influenza peaks.

**Conclusions:**

The proposed approach can be viewed a feasible alternative to forecast ILI in Hong Kong or other countries where ILI has no constant seasonal trend and influenza data resources are limited. The proposed methodology is easily tractable and computationally efficient.

## Introduction

In the past decades, millions of people were struck down by waves of influenza outbreaks. Worldwide, an estimated 3–5 million cases of severe illness occur due to the seasonal influenza, which also cause about 250,000 to 500,000 influenza associated deaths [1]. Furthermore, the 2009 H1N1 pandemic resulted in between 151,700 and 575,400 deaths worldwide only during the first year the virus circulated [2]. Prediction of influenza outbreaks is critical for developing effective strategies for prevention, intervention, and countermeasures, including but not limited to quarantine, vaccination, antiviral campaigns, management of hospital resources, and the United States’ Strategic National Stockpile, which can help to avoid catastrophic consequences and safe lives.

In 2008, Google developed an influenza surveillance web-service, Google Flu Trends (GFT) [3], which aimed to assess current flu activity based on flu-related Google search query [4] [5] [6] [7] [8]. As a result, GFT provided weekly estimates of influenza-like-illness (ILI) rates in 25 countries and several large metropolitan areas in the United States. GFT was discontinued in 2015 but the historical data remain available for public use. Recently, using information from online search engines and social networks to monitor and forecast dynamics of various infectious diseases has become one of the most actively developing research areas in data sciences, including just to name a few, influenza surveillance and forecasting with Twitter [9] [10] [11], Wikipedia [7] [12], UptoDate [13], and Google Correlate [5] [6]. In addition, Polgreen et al. [14] forecast influenza related deaths using search query from Yahoo, while Yuan et al. [15] forecast lab confirmed influenza cases using search query from Baidu. These approaches are typically based on some commonly used linear model, such as, e.g., generalized linear regression (GLM), while model selection is performed, for instance, with least absolute shrinkage and selection operator (LASSO) or penalized regression [5] [6] [13].

However, these studies on utility of nontraditional data sources for flu monitoring and forecasting largely focus on North America and, to some extent, Western Europe [3] [4] [5] [6] [9] [13] [14] [16] [17], while regions of Southeast and East Asia and, particularly, Hong Kong remain substantially under-explored despite the fact that Hong Kong often becomes a regional center of flu outbreaks, such as avian (H5N1) influenza [18], SARS [19], and influenza A (H1N1) [20]. Further, throughout the years, many instances of avian and other non-seasonal flu among humans are also for the first time detected in Hong Kong [21] [22]. Hence, accurately forecasting influenza activity is paramount not only for public health in Hong Kong but also for the worldwide global health preparedness. Many conventional approaches for influenza forecasting developed for North America, however, appear not to be directly applicable to Hong Kong and require various adjustments. For instance, influenza seasons in U.S. exhibit a noticeably more periodic cycle while the influenza season in Hong Kong is relatively aperiodic, which is arguable due to some distinct climate and socio-demographics characteristics of Hong Kong [23] [24]. Some recent yet scarce studies on Hong Kong include Wu et al. [24] who estimated infection attack rate (IAR) and infection-hospitalization probability (IHP) from the serial cross-sectional serologic data and hospitalization data. The estimated IAR and IHP were used to judge the severity in influenza pandemics. Yang et al. [25] used susceptible-infected-recovered (SIR) model with filters to forecast ILI+ rate, which is calculated by both the streams of ILI rate and viral detection rate. Cao et al. [26] developed a dynamic linear model to forecast ILI in Shenzhen (a city neighboring Hong Kong) by using district-level ILI surveillance data and city-level laboratory data. The objective is to generate sensitive, specific, and timely alerts for influenza epidemics in a subtropical city of Southern China. However, these models tend not to deliver sufficiently accurate weekly forecasts of ILI one week or two weeks in advance. Furthermore, to the best of our knowledge, the effectiveness of using Google Trend or other Internet-based sources to forecasting influenza in Hong Kong has not been evaluated yet, and it may be able to produce better forecasting performance.

In this study, we aim to investigate predictive utility of Google search data to forecast the number of new ILI cases (per 1000 consultations) in general outpatient clinics (GOPC) in Hong Kong for one-week and two-weeks ahead. Since GFT was never available in Hong Kong, we use the rate of queries for a small set of flu-related keywords in Google Trend as a proxy for ILI activity. Whenever using flu-related web queries in a predictive platform, one must be cautious of self-excitement effects (i.e., sensitivity to fickle media interest) and lack of calibration of online data [27]. To address these issues, we combine the Google search information in Hong Kong with several offline data sources such previous GOPC flu-related data and current meteorological variables as predictors of future flu activity. To develop ILI forecasts in Hong Kong, we consider three conventional parametric modeling approaches: Generalized Linear Models (GLM), Least Absolute Shrinkage and Selection Operator (LASSO), and Autoregressive integrated moving average (ARIMA). In addition, we also explore utility of deep learning (DL) procedures, namely, Feedforward Neural Networks (FNN), to discover hidden relationships between flu-related online and offline data and exogenous atmospheric variables. The key advantage of DL is its ability to model complex non-linear phenomenon using distributed and hierarchical feature representation [28] [29] [30]. DL is shown to deliver competitive performance in a variety of applications, from computer vision and speech recognition to weather forecasting and transportation network congestion (see, e.g., [28] [31] [32] [33] and references therein). However, to our knowledge, this is the first study that investigates utility of deep learning methodology for surveillance and forecasting of infectious diseases, *i*.*e*. influenza.

We evaluate the forecasting performance of all four models in terms of root mean squared error (RMSE), mean absolute percentage error (MAPE), mean absolute error (MAE), and ability to predict the correct time of seasonal maxima. We find that all four considered models have their own advantages as well as disadvantages during influenza seasons. In order to harness the power of each individual model into a joint unifying framework, we propose using a Bayesian model averaging (BMA) approach, which provides a coherent mechanism of combining the models’ strengths [34] [35]. Our findings indicate that BMA outperforms all the individual models in terms MAPE and RMSE, especially during the periods of highest influenza activity, which is especially important for health risk management.

Our study depicts interesting findings and phenomena. First, we find that DL with FNN is a promising tool for influenza predictive platforms with limited information. Second, BMA appears to be a cohesive unifying framework to combine predictive powers of individual models. Third, even a small and simple set of online web-queries coupled with offline data, which are easy to access and are publicly available in almost every county or city, can deliver reliable forecasts for future flu activity. These findings demonstrate the potential of combining non-traditional flu related data with traditional information sources, to generate computationally efficient and information greedy influenza forecasting, even in regions that exhibit atypical features.

The remainder of the paper is organized in three major sections: Methods, Case Study, and Discussion. In the Methods section, the data sets collected, the study design and the models used in this work are introduced. The next section, compares the forecasting performance of all the models using real data. Finally, we end with discussion and present open questions in the last session.

## Methods

### Data description

#### a. Influenza data

We consider influenza like illness (ILI) data (per 1000 consultations) in general out-patient clinics (GOPC) in Hong Kong. ILI is defined as fever (temperature of 100°F [37.8°C] or greater) and a cough and/or a sore throat without a known cause other than influenza [36]. We extract GOPC’s ILI rates (per 1000 consultations), ILI-GOPC, from week 1, 2011 to week 3, 2016. This information is collected from Hong Kong Centers for Health Protection (CHP): [37]. The ILI rate is reported by a sentinel surveillance network of approximately 50 outpatient clinics. In order to evaluate the forecasting accuracy, we also focus on the influenza seasons during the observed period. According to CDC [36] the influenza season and non-influenza season are separated by the national baseline, which is developed by calculating the mean percentage of patient visits for ILI during non-influenza weeks for the previous three seasons and adding two standard deviations. However, CHP does not provide enough data for us to calculate the national baseline. Therefore, in this study we define influenza seasons as follows: the influenza season is on when there are more than 3 consecutive weeks during which ILI rate is above 5.0 per 1000 consultations.

**Fig 1:**
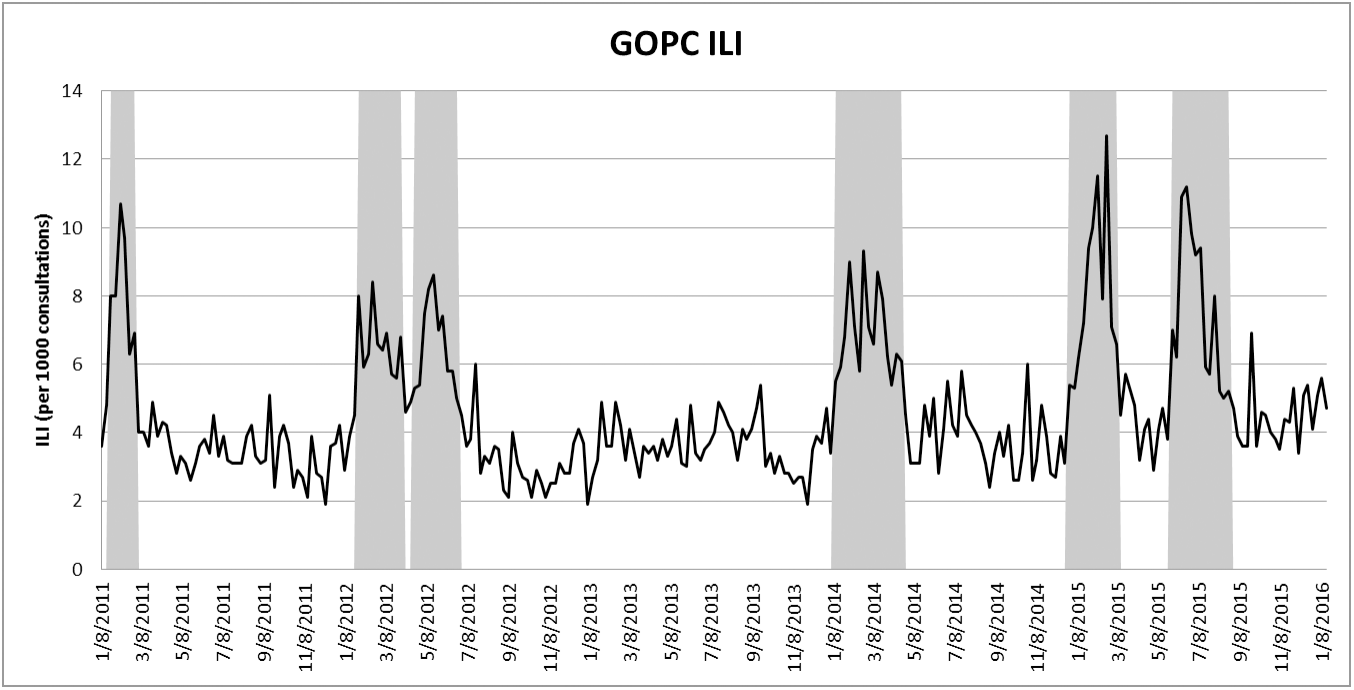
GOPC ILI from week 1, 2011 to week 3, 2016. Influenza seasons are denoted by the shaded areas.

Fig 1 displays the ILI-GOPC for the period 1, 2011–3, 2016. Unlike ILI in U.S., where there is a more clearly defined influenza season each year, there is no sufficiently regular cycle for influenza in Hong Kong. For instance, Fig 1 suggests that there is no influenza in the year 2013; in the year of 2014, there is one influenza season; and in the year of 2012 and 2015, there are two influenza seasons. Besides, the start and duration of influenza seasons largely vary from year to year.

#### b. Google search data

The information from the climate variables is complemented with the search activity in Google for words in relation with influenza. Different keywords have different search frequency and can therefore produce diverse modeling outcomes. Hence, keywords need to be carefully selected to reflect terms most likely related with influenza epidemics. Hong Kong is a city with mix culture, and English as well as Chinese are both used in search engines. However, the search data is limited because of a relatively small representative population (of only 7 million), and Google tools such as Google Correlate are not available in Hong Kong. Based on previous studies of [15] and [38], the following search terms are used as important keywords in this study: cough, cold, flu, h3n2, h7n9, avian flu, fever, 傷風(cold), 咳(cough), 喉嚨痛(sore throat), 感冒(cold), 流感(flu), 發燒(fever),. We collected the normalized frequency of these 13 search terms via Google Trends.

#### c. Meteorological data

Meteorological data have long been recognized as important variables in modeling and predicting influenza activity [16][39][40][41][42][43][44]. The meteorological information considered in this study are daily pressure, absolute max temperature, mean temperature, absolute min temperature, mean dew point, mean relative humidity, mean amount of cloud, rainfall, and bright sunshine. These data are collected by the Hong Kong Observatory [45] and cover the study period from the first week of 2011 to the third week of 2016.

**Remark** Furthermore, data quality control for operational epidemiological modeling and forecasting is a vast and rapidly developing research area in statistical sciences, epidemiology, numerical weather prediction and other disciplines (for more discussion on the current approaches to data quality control see, for example, [46, 47, 48, 49] and references therein). Data quality control constitutes a standalone research topic that falls outside the primary focus of this paper, and hence we leave it as a future extension of the current project.

### Study design

Since the relationship between the ILI-GOPC and Google searches is intrinsically dynamic, in this study we use an adaptive form of out-of-sample forecasting [50]. That is, we use a 104 weeks’ window (i.e., two full years) to train statistical models and then the upcoming weeks to perform out-of-sample forecast validation. A choice of 104 weeks’ window allows to capture the yearly trend as well as seasonal pattern and at the same time has relatively minor assumptions on data availability and consistency of public health protocols. The model parameters are recomputed for each forecast by using only the training data from the previous 104 weeks before the forecasting week. To make a fair and reasonable comparison of different models, all the models use the same adaptive method with a 104 weeks’ window in this study.

### Evaluation metrics

Four metrics are employed to measure the forecast accuracy: the root mean square error (RMSE), mean absolute percentage error (MAPE), and mean absolute error (MAE),. For a series of forecasting values $\left({\hat{y}}_{1},{\hat{y}}_{2},\ldots \ldots ,{\hat{y}}_{n}\right)$ and their corresponding real values (*y*_1_,*y*_2_,……,*y*_*n*_), these metrics are

$$RMSE=\sqrt{\frac{\sum_{i=1}^{n}{\left({\hat{y}}_{i}-y_{i}\right)}^{2}}{n}},$$

$$MAPE=\frac{1}{n}\sum_{i=1}^{n}\left|\frac{{\hat{y}}_{i}-y_{i}}{y_{i}}\right|,$$

$$MAE=\frac{1}{n}\sum_{i=1}^{n}\left|{\hat{y}}_{i}-y_{i}\right|.$$

In addition, as a measure of fit, we consider a sample correlation of estimator $\hat{y}$ and observed *y*. Lower RMSE, MAPE, and MAE imply the better forecasting performance, while the higher the correlation the better.

We also use an ability of each model to predict the time point of maximum influenza activity as an evaluation criterion [17]. As in this study ILI serves as an indicator of influenza activity, an influenza peak is defined as the week of highest ILI rate in each specific influenza season. We then study absolute differences between the observed peak week and the forecasted peak week, produced by each forecasting models. In particular, let *t* be the week of the peak and $\hat{t}$ be the forecasted week of the peak, the week difference is defined as

$$WD=Week\;difference=|t-\hat{t}|,$$

and the smaller WD refers to the more accurate estimation of influenza peaks.

### Statistical methodology

We start from individual models, all of which except deep learning have been considered and widely applied for influenza forecasting (see, for instance, [3] [4] [5] [6] [9] [13] [14] [15] [32] [51] [52] and references therein). We then use Bayesian model averaging to harness predictive power of individual models.

#### a. Generalized Linear Model (GLM)

In this study, we model ILI is a random rate (per 1000 consultations) following a Poisson distribution, and thus consider a Generalized Linear Model (GLM) with a log link (see [53][54] for discussion on GLM). The proposed model also employs autoregressive terms because of an intrinsic time series structure of ILI observations. Yang et al.[6] demonstrate that the recent ILI history is most appropriate to describe the present number of ILI cases, hence, we use only the previous month of ILI as autoregressive terms in GLM. Due to delays of 1–2 weeks in reporting offline ILI information, our model takes the form:

$$\ln\left(y_{t+h}\right)=\mu +\sum_{i=1}^{9}\sum_{j=1}^{7}\alpha_{ij}W_{ij}(t)+\sum_{k=1}^{13}\beta_{k}G_{k}(t)+\sum_{l=2}^{4}\gamma_{l}ln(y_{t-l}),$$

where response *y*_*t*_ is ILI at week *t*,*h* = 1,2,*μ* is the constant intercept, *W*_*ij*_(*t*) is the *i*th meteorological variable on *j*th day of the week *t*, *α*_*ij*_ is the coefficient for *W*_*ij*_(*t*), *i = 1*, *2*, *……*, *9*, *j = 1*, *2*, *……*, *7*; *G*_*k*_(*t*) is the volume of *k*th Google search term, *β*_*k*_ is the coefficient for *G*_*k*_(*t*),*k* = 1,2,……,13; *γ*_*l*_,*l* = 2,3,4 are coefficients for log-transformed previous *y*. The function “glm” is used to fit the GLM model. [55].

#### b. Least absolute shrinkage and selection operator (LASSO)

Penalized linear regression is recently demonstrated to be a promising approach to forecast ILI [5] [6][13]. In particular, penalized linear regression is a generalized linear regression with a certain variable selection procedure. For instance, LASSO, one of the penalization methods, is used to shrink estimation of the regression coefficients in GLM towards zero relative to the maximum likelihood estimates. The objective of this method is to prevent overfitting due to either collinearity of the covariates or high-dimensionality. In our case of ILI forecasting in Hong Kong, if we integrate all the variables into one group (denoted by *X*_*p*_,*p* = 1,2,……,79, which include *W*_*ij*_(*t*), *i = 1*, *2*, *……*, *9*, *j = 1*, *2*, *……*, *7* and *G*_*k*_(*t*),*k* = 1,2,……,13) and (ln(*y*(*t* − *q*)), *q* = 2,3,4), the GLM can be rewritten as

$$\ln\left(y_{t+h}\right)=\mu +\sum_{p=1}^{79}a_{p}X_{p},$$

Then, in a given week *t*, letting $\hat{a}={\left({\hat{a}}_{1},\ldots \ldots ,{\hat{a}}_{79}\right)}^{T}$, the objective of LASSO is to estimate parameters $\left(\hat{a},\hat{\mu }\right)$, that minimize

$$\sum_{t}{\left[\ln\left(y_{t+h}\right)-\mu -\sum_{p=1}^{79}a_{p}X_{p}\right]}^{2}+\lambda \sum_{p}|a_{p}|.$$

LASSO penalty term is based on L_1_ norm, with an idea to shrink coefficients of nonsignificant regressors towards zero, and such regressors are then removed from the model (for more details see, for instance, [56] [57] [58] and [59] and references therein). We use the function “penalized” in penalized R package to fit the LASSO model [60].

#### c. Autoregressive integrated moving average (ARIMA)

As a benchmark, we also consider a Box-Jenkins approach, namely, autoregressive integrated moving average (ARIMA(*p*,*d*,*q*)) model, where *p* is the number of autoregressive (AR) terms, *q* is the order of the non-seasonal moving average (MA) lags, and *d* is the number of non-seasonal differences [61] [62] [63]. The ARIMA has the form:

$$y_{t+h}=\theta_{0}+\sum_{i=1}^{p}\varphi_{i}y_{t+h-i}+\sum_{j=1}^{q}\theta_{j}\varepsilon_{t+h-j}+\varepsilon_{t+h},$$

where *y*_*t*_ is ILI at week *t* and *ε*_*t*_ is white noise random error; *φ*_*i*_ (*i* = 1, 2, …, *p*) and *θ*_*j*_ (*j* = 0,1, 2, …, *q*) are parameters to be estimated using least squares, maximum likelihood etc. On application of ARIMA for flu forecasting see, for instance, [17], [50], [51], [52] and references therein.

Similarly to GLM and LASSO, we re-fit each ARIMA model upon arrival of new observations. Model orders p, q, and d for each ARIMA model are selected from a search over possible model candidates by minimizing the corrected Akaike Information Criterion (AIC) [64]. The function “auto.arima” in the “forecast” R package is used to find the parameters of ARIMA [64][65].

#### d. Deep learning: Feedforward neural networks (FNN)

Deep learning (DL) is a new branch of machine learning, that can be viewed as extension of neural networks (NN) methods and that experience a resurgence following recent advances in computing (see, for instance, [29], [30], and references therein). An appealing property of deep learning is the ability for a model to learn the important features and causal relationships among variables automatically and in a nonparametric data-driven way, instead of relying on manual parametric feature engineering. Deep learning models with multiple layers, have achieved impressive results in many applications such as image classification, natural language processing and drug discovery [66] [67] [68]. However, utility of deep learning procedures for infectious epidemiology remains yet much less investigated [28].

Let us provide a schematic interpretation of a DL idea. Suppose we start from a standard neural network (NN) that consists of many simple, connected processors called neurons, each producing a sequence of real-valued activations. Neural networks have different kind of node layers (input, hidden, and output). Input neurons get activated through sensors perceiving the environment; other neurons get activated through weighted connections from previously active neurons [30]. In application to influenza modeling, the input neurons can be meteorological variables, Google search data, and previously offline-observed ILI data, which constitute the same set of covariates as, for instance, in GLM. The output neurons are then one-week-ahead and two-week-ahead ILI-GOPC. Commonly, NN have one or two hidden layers of neurons. Adding hidden layers tends to increase predictive performance but with a price of increasing computational complexity.

**Fig 2:**
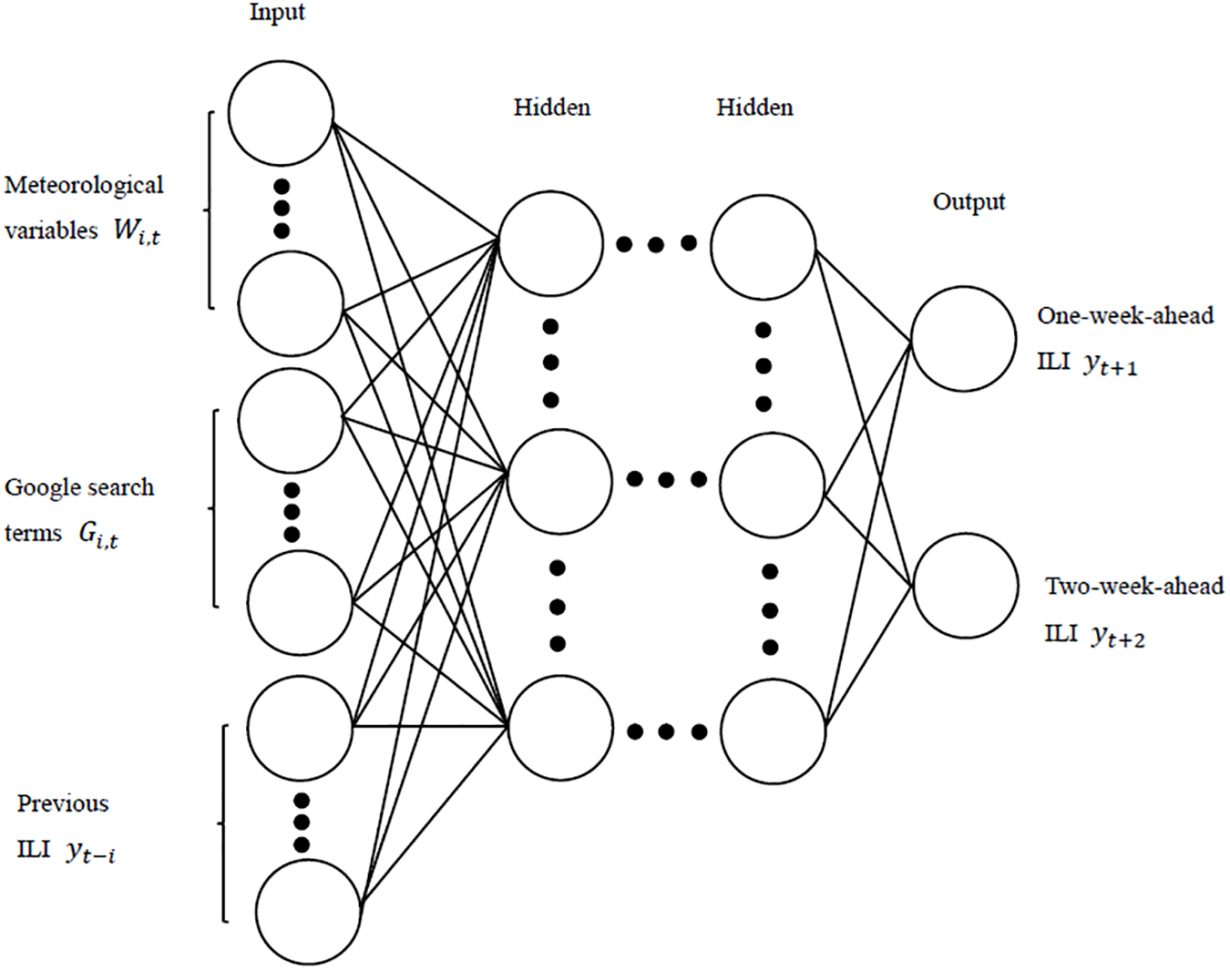
Neural network (NN) with three layers of neurons. The circles denotes neurons in the neural network. The input neurons are the same variables as in GLM and the output neurons are one-week-ahead and two-week-ahead ILI.

In this study, we employ a feedforward (acyclic) NNs (FNN) architecture to train the neural network (see Fig 2) in an unsupervised setting, because of its superior ability of non-linearity to approximate unknown function to any degree of desired accuracy [69]. Our goal is investigate effectiveness of FNN to discover some hidden dependence features among online and offline flu-related data and exogenous regressors and to improve predictive utility of Google search terms.

There exist multiple approaches to select optimal DL architecture (see, e.g. reviews by [70][71][72] and references therein). Conventionally, a number of hidden layers, a number of nodes, and a learning rate are the three primary tuning parameters, that are selected based on a V-fold cross validation, where V is typically five. Other procedures include over-fitting monitoring, pruning and regularization [72]. In addition, the number of hidden nodes should be less than twice the number of input nodes (i.e., in our case less than 158 nodes). In our study, based on the cross validation experiments, we find that the two hidden layers with 50 and 100 nodes, and the learning rate is 0.005 deliver the most competitive performance in terms of mean squared error (MSE). Furthermore, although it is possible to start with a more sophisticated DL architecture and refine it using L_1 or L_2 regularization procedures, such experiments are computationally expensive, and we omit them in this project. The DL feedforward (acyclic) NNs (FNN) architecture is implemented using the open source H2O R package [73]. The function “h2o.deeplearning” is used to train the model in this study.

More information about deep learning and FNN can be found in [29] [30] [70] [71].

#### e. Bayesian model averaging (BMA)

Standard statistical analysis—such as, for example, regression analysis—typically proceeds conditionally on one assumed statistical model. The model is always selected from a basket of statistical models, and the data analyst cannot confirm if it is the best model. In many situations, the best model is just the overall best in terms of accuracy, but this method ignores the uncertainty raised by different answers produced by other feasible models [34] [35]. Therefore, we use Bayesian model averaging (BMA) to address this problem by conditioning, not on a single “best” model, but on the entire ensemble of statistical models first considered. BMA possesses a range of theoretical optimality properties and is shown to deliver competitive predictive performance in a variety of applications [74] [75] [76]. In this paper, the same problem (i.e., whether the best model could be selected by only comparing the evaluation metrics) is faced. Therefore, we introduce BMA to combine all the models to see whether a BMA weighted model combination could produce an enhanced set of ILI forecasts.

Let ${\hat{y}}_{t+h}$ to be the forecasted values, *M*_1_,*M*_2_,…,*M*_*n*_ to be a set of *n* models, and *D* to be the training set. Then the predictive mean of ${\hat{y}}_{t+h},\;h=1,2$ is

$$E[{\hat{y}}_{t+h}\;|D]=\sum_{i=1}^{n}E[{\hat{y}}_{t+h}\;|D,M_{i}]p(M_{i}|D),$$

where *p*(*M*_*i*_|*D*), the posterior probability that the *i-th* model is the true model, is computed as

$$p(M_{i}|D)=\frac{p(D|M_{i})p(M_{i})}{\sum_{j=1}^{n}p(D|M_{j})p(M_{j})},$$

with *p*(*D*|*M*_*i*_) denoting the marginal likelihood, that is calculated as

$$p(D|M_{i})=\int p(D|{\theta_{i},M}_{i}){p(\theta_{i}|M}_{i})d\theta_{i}$$

and *p*(*θ*_*i*_|*M*_*i*_) is the prior density under model *M*_*i*_.

For more detailed discussion on BMA see, for instance, [34], [35] and references therein. The function “bicreg” in the BMA R package is used to run the BMA model [77].

## Case study

We now evaluate the proposed approaches and data by adaptively forecasting ILI-GOPC for the latest two years (i.e., week 4, 2014 to week 3, 2016). We first use four individual models, GLM, ARIMA, LASSO and DL with FNN, to forecast ILI in this period. When using LASSO, we set λ = 10, and 25 terms are selected, on average, each week. The previous ILI incidences are always excluded, and different meteorological and Google search terms are included in each fitting. S3 Table shows the parameters p, d, and q selected in ARIMA. FNN uses two hidden layers with 50 and 100 nodes, and the learning rate is 0.005. Table 1 shows the forecasting performance of these four models for the whole forecasting period and the seasons on influenza activity (i.e., “influenza seasons”). In particular, according to our definition, there are three influenza seasons in the forecasting period: (1) Influenza Season I: January 25, 2014 to April 19, 2014; (2) Influenza Season II: December 27, 2014 to March 7, 2015; (3) Influenza Season III: May 30, 2015 to August 22, 2015. Correspondingly, there exist three influenza spikes: Peak I (week 8, 2014), Peak II (week 8, 2015), and Peak III (week 25, 2015). Table 2 displays the week difference of influenza peaks.

**Table 1:** Comparison of GLM, ARIMA, LASSO, DL with FNN, and BMA for estimation of GOPC-ILI in terms of the evaluation metrics.

One-week-ahead forecasting								
	Whole period				Influenza seasons			
Method	RMSE	MAPE	MAE	Corr.	RMSE	MAPE	MAE	Corr.
GLM	1.97	25.9%	1.39	**0.65**	2.77	25.7%	2.00	0.47
ARIMA	2.14	28.6%	1.53	0.47	2.91	30.6%	2.36	0.07
LASSO	1.84	28.2%	1.45	0.57	2.43	24.1%	1.95	0.35
DL with FNN	1.73	25.4%	1.30	0.63	2.23	**19.8%**	1.66	0.50
BMA	**1.53**	**24.5%**	**1.23**	**0.73**	**1.95**	21.7%	**1.65**	**0.61**
Two-week-ahead forecasting								
	Whole period				Influenza seasons			
	RMSE	MAPE	MAE	Corr.	RMSE	MAPE	MAE	Corr.
GLM	2.14	26.1%	1.47	0.60	3.33	35.4%	2.40	0.37
ARIMA	2.43	33.3%	1.81	0.30	3.34	35.5%	2.77	-0.17
LASSO	1.84	27.2%	1.38	0.59	2.44	23.2%	1.84	0.30
DL with FNN	1.69	**26.0%**	1.35	0.67	2.22	23.6%	1.87	**0.73**
BMA	**1.68**	28.4%	**1.33**	**0.69**	**1.90**	**21.7%**	**1.56**	0.60

**Table 2:** Week difference (WD) of influenza peaks.

	One-week-ahead forecasting				Two-week-ahead forecasting			
Method	Peak I	Peak II	Peak III	Mean	Peak I	Peak II	Peak III	Mean
GLM	2	2	3	2.3	1	0	2	**1.0**
ARIMA	0	3	3	2.0	1	4	4	3.0
LASSO	1	1	2	1.3	2	1	4	2.3
DL with FNN	0	0	1	**0.3**	1	2	0	**1.0**
BMA	1	1	0	0.7	2	0	2	1.3

Table 1 indicates that the DL with FNN is the most competitive approach in terms of RMSE, MAPE, and MAE for one week ahead forecasting. The DL with FNN also yields highly competitive results for two weeks ahead forecasting in terms of all evaluation metrics (except of MAPE and MAE where the DL with FNN is the second best to LASSO). Table 2 suggests that for one-week-ahead forecasting, the DL with FNN is the best model with the lowest mean of WD. In particular, for Peaks I and II, the DL with FNN accurately predict the peak week (i.e., delivered WD is 0), while for Peak III, there is only one week difference between the forecasted and observed series (i.e., WD is 1). For two-week-ahead forecasting, both the DL with FNN and GLM are the two best models. The mean week differences of these models are both 1 week, which is slightly higher than that of one-week-ahead forecasting. Hence, we can conclude that the DL with FNN tends to the preferred approach for influenza forecasting in Hong Kong.

**Fig 3:**
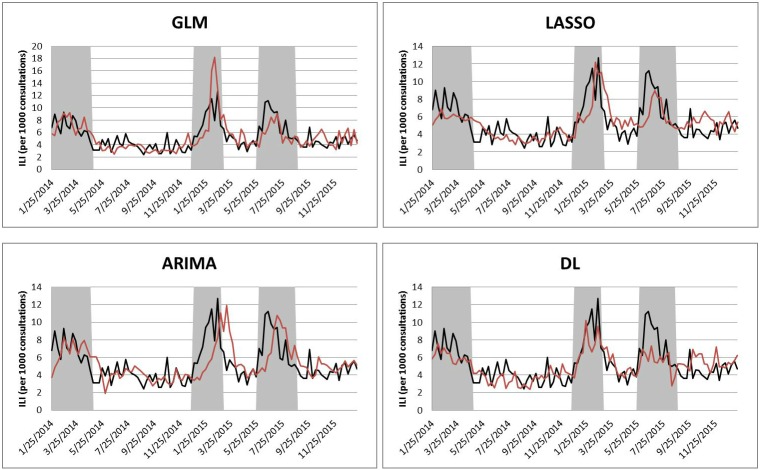
Results of one-week-ahead forecasting with four individual approaches. In each figure, the black line denotes the observed ILI and the red line denotes the estimated ILI from each model. The shaded areas denote the influenza seasons.

**Fig 4:**
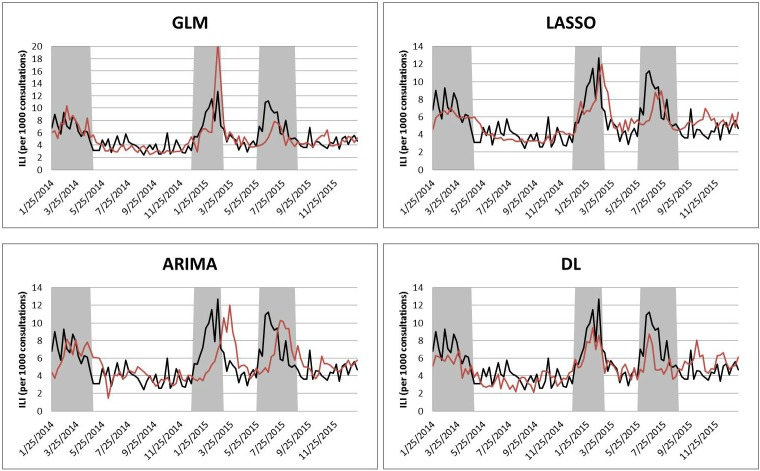
Results of two-week-ahead forecasting with four individual approaches. In each figure, the black line denotes the observed ILI and the red line denotes the estimated ILI from each model. The shaded areas denote the influenza seasons.

Figs 3 and 4 show the forecasting results of GLM, LASSO, and DL with FNN. Although the DL with FNN produces the overall best forecasting result, it does not perform well during all the three influenza seasons where the forecasted values of deep learning always underestimate the observed values. The forecasted ILI during influenza seasons produced by ARIMA are close to observed values, but there are peak delays, especially in Influenza Season II and III. GLM sometimes overestimates the number of observations (Influenza Season II), and sometimes underestimates them (Influenza Season III). LASSO also underestimates the ILI values (Influenza Season I and III). Both GLM and LASSO have delays to forecast the start of influenza seasons.

Therefore, we use BMA to combine all these four models in order to develop a more accurate predictive tool for ILI in Hong Kong. BMA offers a comprehensive approach to weight (or score) each model based on its recent performance such that the model weights (scores) vary over time. This in turn allows to better capture intrinsically dynamic relationships among all the forecasting approaches. In order to discover the best length of window, we evaluate lengths of window from 8 to 26 weeks. For one-week-ahead forecasting, when the length of window is 18 weeks, BMA produces the most accurate forecasted values during influenza seasons (S1 Table). A 17 weeks’ sliding window can lead to accurate performance for two-week-ahead forecasting (S2 Table).

**Fig 5:**
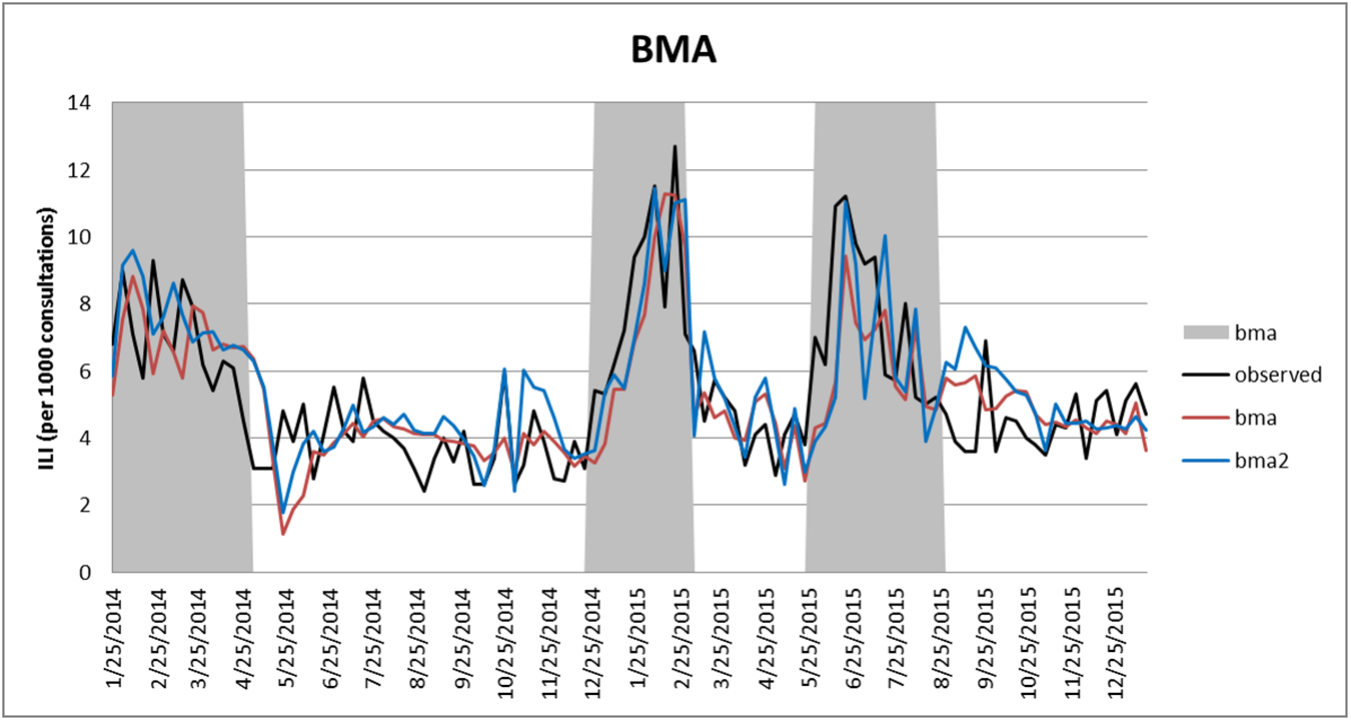
Results of BMA for one-week-ahead forecasting (18 weeks’ window) and two-week-ahead forecasting (17 weeks’ window). The black line denotes the observed ILI, the red line denotes the one-week-ahead estimated ILI, and the blue line denotes the two-week-ahead estimated ILI. The shaded areas denote the influenza seasons.

Fig 5 presents the results of BMA when the length of sliding windows is 18 weeks and 17 weeks for one-week- and two-week-ahead forecasting, respectively. BMA takes advantages of and overcomes the shortcomings of each model, especially during the active influenza seasons. In particular, the BMA forecasts more closely follow the observed ILI curves than any of the forecasts based on the individual models (Figs 3 and 4, and supplementary S1 and S2 Tables). However, in terms of peak week prediction, when using 18 weeks’ window to forecast ILI-GOPC one week ahead, WD for BMA in Peak I, II, and III are 1, 1, and 0, respectively; when using 17 weeks’ window to forecast ILI-GOPC two weeks ahead, WD for BMA in Peak I, II, and III are 2, 0, and 2, respectively. Hence, although BMA improves the overall predictive fit in terms MAPE and RMSE, it does not improve the forecasting accuracy of influenza peaks, and the DL with FNN still remains the most competitive method to forecast locations of influenza peaks.

## Discussion

In this study we evaluate a predictive utility of Google flu-related searches in Hong Kong, coupled with offline influenza data and exogenous meteorological information. We consider a number of conventional parametric forecasting models such as GLM, penalized regression, and ARIMA, as well as a data-driven nonparametric deep learning approach with a feedforward (acyclic) neural networks (DL with FNN). Despite the fact that deep learning is shown to deliver competitive performance in modeling and forecasting a wide range of complex natural phenomena and disparate social systems, its utility for epidemiological studies is yet underexplored. To our knowledge, this is the first study that employs deep learning methodology for surveillance and forecasting of infectious diseases and explores its predictive utility of DL in application to influenza. Furthermore, to integrate and harness predictive power of individual models, we also employ a unified framework of Bayesian Model Averaging. Our results suggest a number of remarkable findings. First, indeed, even a small set of flu-related Google search queries, coupled with offline data, can yield reasonable forecasts of future flu activity both in terms of the ILI curve and location of outbreak spike. Second, among all individual models, the data-driven deep learning (DL) method with FNN is certainly a preferred candidate. Finally, we find that neither model can be considered as universally best, i.e., the principle “one size fits all” is not appropriate, that is, although BMA is a very useful tool to improve forecasts of the ILI curve, the DL with FNN is still the best for predicting the outbreak peak location.

In general, our proposed approach can be a feasible alternative to forecast ILI in Hong Kong or other countries where ILI has no constant seasonal trend and external data is limited. The method could produce accurate ILI forecasts and provide policymakers with sufficient time to enhance health risk management strategies.

In the future we plan to evaluate our approach in application to other geographical regions, with a particular focus, on substantially less investigated developing countries in Southeast and East Asia, and to incorporate local socio-demographic information and networks of everyday social contacts. To assess a hierarchical social communication structure of contacts, we can use various offline information sources, such as census, school enrollment and public transportation data (see, e.g., [78], [79]) and georeferenced online data such as Meetup and Twitter [80]. We also plan to extend our approach to a setting of probabilistic forecasting of flu, that is, to develop a full predictive density of future flu scenarios.

## Supporting information

S1 Table Comparison of one-week-ahead forecasting accuracy of BMA with different window sizes. (DOCX)

S2 Table Comparison of two-week-ahead forecasting accuracy of BMA of different window sizes. (DOCX)

S3 Table The parameters p, d, and q in ARIMA. (DOCX)
