# Supplementary material for: Forecasting influenza in Hong Kong with Google search queries and statistical model fusion
Source: PLoS One. 2017 May 2;12(5):e0176690. doi: 10.1371/journal.pone.0176690 (PMC5413039; doi:10.1371/journal.pone.0176690)
Supplement: S1 Table — (DOCX) [file pone.0176690.s001.docx]

S1 Table. Comparison of one-week-ahead forecasting accuracy of BMA with different window sizes

|  | Whole period | | | | Influenza season | | | |
| --- | --- | --- | --- | --- | --- | --- | --- | --- |
| Length of window | RMSE | MAPE | MAE | Correlation | RMSE | MAPE | MAE | Correlation |
| 8 | 1.76 | 25.5% | 1.32 | 0.69 | 2.36 | 28.9% | 2.02 | 0.43 |
| 9 | 1.67 | 24.4% | 1.27 | 0.71 | 2.24 | 27.8% | 1.96 | 0.47 |
| 10 | 1.66 | 24.9% | 1.29 | 0.71 | 2.14 | 26.2% | 1.88 | 0.50 |
| 11 | 1.65 | 25.4% | 1.31 | 0.70 | 2.1 | 25.3% | 1.87 | 0.51 |
| 12 | 1.66 | 25.4% | 1.30 | 0.69 | 2.1 | 24.1% | 1.79 | 0.50 |
| 13 | 1.62 | 25.3% | 1.29 | 0.70 | 2.09 | 23.1% | 1.75 | 0.51 |
| 14 | 1.61 | 24.9% | 1.28 | 0.70 | 2.05 | 22.9% | 1.75 | 0.52 |
| 15 | 1.6 | 25.0% | 1.28 | 0.70 | 2.07 | 22.8% | 1.75 | 0.52 |
| 16 | 1.55 | 25.0% | 1.27 | 0.72 | 2.01 | 23.1% | 1.74 | 0.56 |
| 17 | 1.57 | 25.0% | 1.27 | 0.71 | 2.05 | 22.7% | 1.72 | 0.56 |
| 18 | **1.53** | 24.5% | 1.23 | **0.73** | **1.95** | 21.7% | **1.65** | **0.61** |
| 19 | 1.6 | 24.8% | 1.26 | 0.70 | 2.08 | 22.2% | 1.71 | 0.57 |
| 20 | 1.63 | 25.0% | 1.28 | 0.68 | 2.15 | 22.6% | 1.76 | 0.54 |
| 21 | 1.64 | 25.5% | 1.30 | 0.68 | 2.16 | 22.9% | 1.79 | 0.54 |
| 22 | 1.61 | 24.9% | 1.28 | 0.69 | 2.14 | 22.8% | 1.78 | 0.56 |
| 23 | 1.57 | 24.6% | 1.26 | 0.70 | 2.07 | 22.3% | 1.75 | 0.57 |
| 24 | 1.57 | 24.5% | 1.26 | 0.70 | 2.09 | 22.5% | 1.75 | 0.56 |
| 25 | 1.55 | 24.3% | 1.24 | 0.71 | 2.05 | 21.9% | 1.71 | 0.58 |
| 26 | 1.55 | **23.9%** | **1.22** | 0.71 | 2.05 | **21.6%** | 1.70 | 0.58 |
