# Supplementary material for: Forecasting influenza in Hong Kong with Google search queries and statistical model fusion
Source: PLoS One. 2017 May 2;12(5):e0176690. doi: 10.1371/journal.pone.0176690 (PMC5413039; doi:10.1371/journal.pone.0176690)
Supplement: S2 Table — (DOCX) [file pone.0176690.s002.docx]

S2 Table. Comparison of two-week-ahead forecasting accuracy of BMA of different window sizes

|  | Whole period | | | | Influenza season | | | |
| --- | --- | --- | --- | --- | --- | --- | --- | --- |
| Length of window | RMSE | MAPE | MAE | Correlation | RMSE | MAPE | MAE | Correlation |
| 8 | 2.06 | 29.1% | 1.47 | 0.62 | 2.97 | 32.1% | 2.20 | 0.34 |
| 9 | 2.08 | 28.8% | 1.48 | 0.60 | 2.97 | 30.4% | 2.19 | 0.38 |
| 10 | 2.01 | 28.9% | 1.48 | 0.62 | 2.84 | 30.1% | 2.18 | 0.41 |
| 11 | 1.86 | 28.0% | 1.41 | 0.65 | 2.56 | 28.1% | 2.02 | 0.44 |
| 12 | 1.89 | 28.2% | 1.40 | 0.63 | 2.55 | 26.8% | 1.93 | 0.44 |
| 13 | 1.85 | **26.5%** | 1.31 | 0.64 | 2.52 | 25.4% | 1.84 | 0.44 |
| 14 | 1.69 | 26.7% | 1.30 | 0.68 | 2.14 | 23.2% | 1.68 | 0.50 |
| 15 | 1.75 | 28.0% | 1.35 | 0.66 | 2.17 | 23.6% | 1.71 | 0.49 |
| 16 | 1.78 | 29.0% | 1.39 | 0.65 | 2.18 | 24.0% | 1.74 | 0.49 |
| 17 | **1.68** | 28.4% | **1.33** | **0.69** | **1.9** | **21.7%** | **1.56** | **0.60** |
| 18 | 1.72 | 28.8% | 1.36 | 0.67 | 1.96 | 22.6% | 1.63 | 0.58 |
| 19 | 1.74 | 29.0% | 1.37 | 0.66 | 2.02 | 22.6% | 1.64 | 0.58 |
| 20 | 1.72 | 28.6% | 1.36 | 0.66 | 2.04 | 22.7% | 1.66 | 0.57 |
| 21 | 1.71 | 28.7% | 1.35 | 0.67 | 1.97 | 21.8% | 1.60 | 0.59 |
| 22 | 1.7 | 28.1% | 1.33 | 0.67 | 1.99 | 22.1% | 1.62 | 0.58 |
| 23 | 1.7 | 28.6% | 1.35 | 0.67 | 1.98 | 22.0% | 1.62 | 0.58 |
| 24 | 1.7 | 28.4% | 1.35 | 0.67 | 1.98 | 22.0% | 1.63 | 0.58 |
| 25 | 1.7 | 28.6% | 1.36 | 0.66 | 1.98 | 22.0% | 1.64 | 0.57 |
| 26 | 1.7 | 28.4% | 1.36 | 0.66 | 2.00 | 22.2% | 1.66 | 0.57 |
