# Supplementary material for: Forecasting influenza in Hong Kong with Google search queries and statistical model fusion
Source: PLoS One. 2017 May 2;12(5):e0176690. doi: 10.1371/journal.pone.0176690 (PMC5413039; doi:10.1371/journal.pone.0176690)
Supplement: S3 Table — (DOCX) [file pone.0176690.s003.docx]

S3 Table. The parameters p, d, and q in ARIMA

| No. | p | D | q | No. | p | d | q | No. | p | d | q |
| --- | --- | --- | --- | --- | --- | --- | --- | --- | --- | --- | --- |
| 1 | 1 | 0 | 1 | 46 | 2 | 1 | 0 | 91 | 0 | 1 | 2 |
| 2 | 0 | 1 | 1 | 47 | 0 | 1 | 1 | 92 | 0 | 1 | 1 |
| 3 | 0 | 1 | 1 | 48 | 2 | 1 | 0 | 93 | 0 | 1 | 2 |
| 4 | 0 | 1 | 1 | 49 | 1 | 1 | 3 | 94 | 0 | 1 | 2 |
| 5 | 0 | 1 | 1 | 50 | 2 | 1 | 0 | 95 | 0 | 1 | 2 |
| 6 | 0 | 1 | 1 | 51 | 2 | 1 | 0 | 96 | 0 | 1 | 2 |
| 7 | 0 | 1 | 1 | 52 | 0 | 1 | 1 | 97 | 0 | 1 | 2 |
| 8 | 0 | 1 | 1 | 53 | 0 | 1 | 1 | 98 | 2 | 0 | 2 |
| 9 | 0 | 1 | 1 | 54 | 0 | 1 | 1 | 99 | 2 | 0 | 3 |
| 10 | 0 | 1 | 1 | 55 | 0 | 1 | 1 | 100 | 2 | 0 | 2 |
| 11 | 0 | 1 | 1 | 56 | 3 | 1 | 0 | 101 | 2 | 0 | 2 |
| 12 | 0 | 1 | 1 | 57 | 2 | 1 | 0 | 102 | 2 | 0 | 3 |
| 13 | 1 | 1 | 2 | 58 | 1 | 1 | 3 | 103 | 1 | 0 | 3 |
| 14 | 2 | 1 | 0 | 59 | 0 | 1 | 1 | 104 | 2 | 0 | 2 |
| 15 | 0 | 1 | 1 | 60 | 0 | 1 | 1 | 105 | 2 | 0 | 2 |
| 16 | 0 | 1 | 1 | 61 | 0 | 1 | 1 | 106 | 2 | 0 | 2 |
| 17 | 0 | 1 | 1 | 62 | 2 | 1 | 0 | 107 | 2 | 0 | 2 |
| 18 | 0 | 1 | 1 | 63 | 2 | 1 | 0 | 108 | 1 | 2 | 0 |
| 19 | 0 | 1 | 1 | 64 | 2 | 1 | 0 | 109 | 1 | 1 | 0 |
| 20 | 0 | 1 | 1 | 65 | 2 | 1 | 0 | 110 | 1 | 1 | 0 |
| 21 | 0 | 1 | 1 | 66 | 0 | 1 | 1 | 111 | 1 | 1 | 0 |
| 22 | 0 | 1 | 1 | 67 | 0 | 1 | 1 | 112 | 1 | 1 | 0 |
| 23 | 0 | 1 | 1 | 68 | 0 | 1 | 1 | 113 | 1 | 1 | 0 |
| 24 | 0 | 1 | 1 | 69 | 0 | 1 | 1 | 114 | 1 | 1 | 0 |
| 25 | 0 | 1 | 1 | 70 | 0 | 1 | 1 | 115 | 0 | 1 | 2 |
| 26 | 1 | 1 | 1 | 71 | 0 | 1 | 1 | 116 | 0 | 1 | 2 |
| 27 | 0 | 1 | 1 | 72 | 0 | 1 | 1 | 117 | 1 | 1 | 0 |
| 28 | 0 | 1 | 1 | 73 | 0 | 1 | 1 | 118 | 1 | 1 | 0 |
| 29 | 1 | 1 | 2 | 74 | 0 | 1 | 1 | 119 | 1 | 1 | 0 |
| 30 | 3 | 1 | 0 | 75 | 0 | 1 | 1 | 120 | 2 | 0 | 2 |
| 31 | 2 | 1 | 0 | 76 | 0 | 1 | 1 | 121 | 2 | 0 | 2 |
| 32 | 2 | 1 | 3 | 77 | 0 | 1 | 1 | 122 | 2 | 0 | 3 |
| 33 | 3 | 1 | 5 | 78 | 1 | 0 | 1 | 123 | 2 | 0 | 2 |
| 34 | 0 | 1 | 1 | 79 | 1 | 0 | 1 | 124 | 2 | 0 | 2 |
| 35 | 2 | 1 | 0 | 80 | 1 | 0 | 1 | 125 | 2 | 0 | 2 |
| 36 | 2 | 1 | 0 | 81 | 3 | 0 | 0 | 126 | 2 | 0 | 2 |
| 37 | 0 | 1 | 1 | 82 | 1 | 0 | 1 | 127 | 2 | 0 | 2 |
| 38 | 0 | 1 | 1 | 83 | 1 | 0 | 1 | 128 | 2 | 0 | 2 |
| 39 | 1 | 0 | 1 | 84 | 3 | 0 | 0 | 129 | 2 | 0 | 2 |
| 40 | 1 | 0 | 1 | 85 | 1 | 0 | 1 | 130 | 2 | 0 | 2 |
| 41 | 3 | 0 | 0 | 86 | 1 | 0 | 1 | 131 | 2 | 0 | 2 |
| 42 | 2 | 1 | 0 | 87 | 1 | 0 | 1 | 132 | 2 | 0 | 2 |
| 43 | 0 | 1 | 1 | 88 | 4 | 0 | 1 | 133 | 3 | 0 | 2 |
| 44 | 0 | 1 | 1 | 89 | 5 | 2 | 1 |  |  |  |  |
| 45 | 2 | 1 | 2 | 90 | 2 | 1 | 2 |  |  |  |  |
